# The Ecraseur in Ovariotomy

**Published:** 1858-05

**Authors:** 


THE ECRASEUR IN OVARIOTOMY.—Dr. John L. Atlee, of Lancaster, Pa., on the 23d of March, removed a large multilocular ovarian tumor from a lady sixty-one years of age, by means of the écraseur. He cast the instrument around the pedicle, which was one inch in length by four in breadth, and highly vascular, and succeeded in removing it in six minutes and a half without the loss of a drop of blood. The external wound was closed by silver sutures, and the woman made a rapid recovery.

A full report of the case will appear in the next number of this journal.

